# Reproductive outcomes from ten years of elective oocyte cryopreservation

**DOI:** 10.1007/s00404-022-06711-0

**Published:** 2022-08-21

**Authors:** Lorraine S. Kasaven, Benjamin P. Jones, Carleen Heath, Rabi Odia, Joycelia Green, Aviva Petrie, Srdjan Saso, Paul Serhal, Jara Ben Nagi

**Affiliations:** 1 Department of Gynaecology, Queen Charlotte’s and Chelsea Hospital, Imperial College London NHS Trust, Du Cane Road, London, W12 OHS UK; 2 Department of Cancer and Surgery, Imperial College London, London, W12 0NN UK; 3 Department of Cutrale Perioperative and Ageing Group, Imperial College London, London, W12 0NN UK; 4 Centre for Reproductive and Genetic Health, Great Portland Street, London, W1W 5QS UK; 5 Biostatistics Unit, UCL Eastman Dental Institute, University College London, 256 Grays Inn Road, London, WC1X 8LD UK

**Keywords:** Anti-Müllerian hormone, Fertility preservation, Elective oocyte cryopreservation, Vitrification, Livebirth rate

## Abstract

**Research question:**

To assess the relationship between the number of oocytes retrieved during elective oocyte cryopreservation (EOC) cycles with various clinical, biochemical, and radiological markers, including age, body mass index (BMI), baseline anti-Müllerian hormone (AMH), antral follicle count (AFC), Oestradiol level (E2) and total number of follicles ≥ 12 mm on the day of trigger. To also report the reproductive outcomes from women who underwent EOC.

**Methods:**

A retrospective cohort of 373 women embarking on EOC and autologous oocyte thaw cycles between 2008 and 2018 from a single London clinic in the United Kingdom.

**Results:**

483 stimulation cycles were undertaken amongst 373 women. The median (range) age at cryopreservation was 38 (26–47) years old. The median numbers of oocytes retrieved per cycle was 8 (0–37) and the median total oocytes cryopreserved per woman was 8 (0–45). BMI, E2 level and number of follicles ≥ 12 mm at trigger were all significant predictors of oocyte yield. Multivariate analysis confirmed there was no significant relationship between AFC or AMH, whilst on univariate analysis statistical significance was proven. Thirty six women returned to use their cryopreserved oocytes, of which there were 41 autologous oocyte thaw cycles undertaken. There were 12 successful livebirths achieved by 11 women. The overall livebirth rate was 26.8% per cycle. No livebirths were achieved in women who underwent EOC ≥ 40 years old, and 82% of all livebirths were achieved in women who had done so between 36 and 39 years old.

**Conclusion:**

Clinical, biochemical and radiological markers can predict oocyte yield in EOC cycles. Reproductive outcomes are more favourable when cryopreservation is performed before the age of 36, with lower success rates of livebirth observed in women aged 40 years and above.

## What does this study add to the clinical work

**Table:** 

Women should be encouraged to undergo elective oocyte cryopreservation before the age of 36 as a preventative measure of age related fertility decline. When the procedure is performed above this age, the chances of successful livebirth are significantly reduced.	

## Introduction

The development of oocyte vitrification, with success rates now similar to fresh cycles [1], has enabled the opportunity to electively cryopreserve oocytes prior to the physiological decline in oocyte quality and quantity. Referred to herein as elective oocyte cryopreservation (EOC), it negates the age-associated deterioration in reproductive potential, but remains a controversial option owing to the multitude of ethical, legal, economic and obstetric related issues that it provokes [2]. Whilst some have little alternative option, such as single women approaching their late-thirties who desire biologically related offspring [3], other indications include delaying childbearing for furthering of education, or to focus on achieving career goals [4]. Despite the theoretical benefit of preserving reproductive potential through EOC, it does not guarantee against involuntary childlessness [5]. Oocytes can be lost during the thaw process, or following unsuccessful embryo transfer,  potentially exhausting the supply of cryopreserved oocytes. Replenishment may subsequently be difficult in the same woman at a later age, owing to the inevitable physiological depletion in ovarian reserve [6]. Therefore, at the time of EOC, it is important to optimise the number of oocytes retrieved for cryopreservation to improve the probability of a successful livebirth [7].

Various characteristics are associated with oocyte yield, such as the age at the time of oocyte retrieval [8]. This encapsulates the very principle of EOC, and directly relates to the progressive depletion in primordial follicles and reduced neuroendocrine response to ovarian stimulation. This clinically manifests with poorer oocyte quantity and quality, as demonstrated by a multivariate analysis to control for other confounding variables [9]. Contrarily, increased BMI is also associated with a reduction in oocyte yield [10].

The most accurate markers of ovarian reserve considered for prediction of oocyte yield following controlled ovarian stimulation (COS), are antral follicle count (AFC) and anti-Müllerian hormone (AMH) [11]. Endocrine markers including Oestradiol (E2), a measure of granulosa cell function has also been used [12]. Furthermore, elevated levels of follicle stimulating hormone (FSH) on day 2–3 of a cycle is associated with a significant reduction in yield, when compared to women with lower FSH of the same age [13]. Radiologically, a follicular volume between 13 and 23 mm/1–6 mL at the time of ovulation trigger is also associated with optimal yields [14].

The aim of this study was to assess the relationship between oocyte yield with clinical, biochemical and radiological markers including age, BMI, AMH, AFC, E2 and total number of follicles ≥ 12 mm on the day of trigger. The secondary aim was to report the reproductive outcomes following EOC in one of the largest reported cohorts in the United Kingdom (UK) thus far.

## Materials and methods

### Selection criteria

This retrospective cohort included all women who approached the Centre for Reproductive and Genetic Health (CRGH) in London, UK for EOC over a ten-year period between 2008 and 2018.

### Clinical protocols

The clinical protocol described herein was applied consistently throughout the duration of the study. An initial assessment was carried out, which included a blood test for AMH (Roche Assay, UK) FSH, E2 and an ultrasound for AFC performed between days 1–4 of the menstrual cycle. The ovarian stimulation protocol was individualised depending on age, AFC, AMH and BMI. Ovarian stimulation using an antagonist protocol consisting of the gonadotropins Fostimon (Urofollitropin) (Pharmasure, UK) and Merional (Menopur) (Pharmasure, UK), was commenced on the third day of the menstrual cycle. From day 6 onwards, the gonadotropin dose was adjusted according to the E2 levels and ultrasound evidence of stimulation. On day 7 of the cycle, and/or once the leading follicle was ≥ 14 mm in diameter, Cetrorelix (Cetrotide) (Merck Serono, Germany) 0.25 mg was commenced. The choice of trigger was individualised after assessment of age, baseline AFC and AMH, number of follicles and E2 levels on the day of trigger. Women considered low responders (those with a diminished ovarian reserve as per the Bologna criteria), were administered with either Pregnyl (Organon, Netherlands), a human chorionic gonadotropin (hCG) trigger, or dual trigger with Pregnyl 1,500 IU and Suprefact (Sanofi-Aventis, Germany) 1 ml, a gonadotropin releasing hormone analogue (GnRH). Women considered normal or high responders (i.e. women with the following risk factors for ovarian hyperstimulation syndrome (OHSS): > 20 follicles measuring > 12 mm on the day of trigger and an E2 level ≥ 20,000 pmol/L) were given Suprefact only. Oocyte maturation was triggered once the mean diameter of ≥ 3 follicles was ≥ 18 mm. In the event women were at high risk of OHSS, Suprefact 1 ml was administered and the LH level was measured 8–12 h later. Oocyte retrieval was scheduled 37 h after the ovulation trigger. Denudation was carried out 39 h post the trigger injection and metaphase II oocytes were vitrified immediately thereafter.

All thaw cycles included within our study refer to the thawing of oocytes which were cryopreserved initially at CRGH, undergoing the vitrification method. Vitrification at room temperature (24–26 °C) took place in two steps. Firstly, once oocytes were denuded they were moved for 12–15 min within a solution containing 7.5% dimethyl sulfoxide-D6 (DMSO) and 7.5% ethylene glycerol (EG). Oocytes achieving full recovery to normal size were then transferred to a vitrification solution consisting of 15% DMSO and EG plus 0.5 M. sucrose for 60 s. Secondly, oocytes were then transferred via Cryotop (Kitazato, Japan) straws at fast rate into liquid nitrogen for storage, within a minimum volume of vitrification solution. Six welled dishes adjacent to three welled plates from Cryotech (Kitazato, Japan) were used, containing solutions warmed at 37° with various concentrations of sucrose (1.0. 0.5 and 0.0 M.). The vitrified oocytes within the Cryotop straws were then removed from storage and moved to liquid nitrogen, within 0.7 ml 1.0 M. of sucrose, followed by 3–5 min within 50 μl of varying decreasing sucrose concentrations at room temperature (24–26 °C). Oocytes with full recovery were then transferred for 2 h into culture (Origio Sequential Fert, CooperSurgical Fertility Solutions, Denmark), before undergoing intracytoplasmic sperm injection (ICSI).

The oocytes were assessed 16–18 h post ICSI for the presence of pronuclei and were separated according to the number of pronuclei present. Oocytes displaying abnormal pronuclei numbers (zero, one and more than three) were either discarded or kept in a separate dish of cleavage medium (Vitrolife, Sweden). Oocytes displaying 2 pronuclei were either moved to a fresh dish of cleavage medium (Vitrolife), while the two-step culture system was in effect, or single-step culture media (SAGE, Origio, Denmark). The embryos were either moved to Blastocyst medium (Vitrolife), or a fresh dish of single-step media (SAGE) on day 3 in order to be cultured to the blastocyst stage, until day 5 or 6 post insemination. Any embryos that successfully formed a blastocyst on day 5 or 6 of culture (exhibiting the presence of clear inner cell mass and trophectoderm cell lines) were selected for vitrification.

Blastocyst vitrification took place at 37 °C. Multiple 5-well Nunc dishes were used to warm 1 × 0.5 ml wells of V1 per blastocyst, 1 × 0.5 ml well of V2 and 1 × 1 ml well of V3 media solutions (Blastocyst Vitrification Kit, Sydney IVF) to 37 °C a minimum of 1 h prior to use. Blastocysts were isolated into micro-drops of warm Hepes media (ThermoFisher, USA) under oil, assessed at 200×magnification and graded as per the CRGH in-house grading criteria (adapted Cornell). Blastocoelic cavity collapse was then initiated by a single laser shot to the junction of two outermost trophectodermal cells, before the blastocysts were transferred to individual wells of the warmed V1 media (up to 4 blastocysts per dish).

Vitrification was conducted by moving a single blastocyst from V1 to V2 media for 2 min, followed by thorough washing through 3 drops of V3 media for 30–35 s. The blastocyst was then loaded rapidly onto a Cryolock with minimal volume of vitrification solution, and plunged into liquid nitrogen before placing a protective cap over the loading strip. Once blastocysts were frozen, women embarked on a single elective embryo transfer and a medicated frozen embryo transfer was performed [15]. All patients were advised to perform a urinary pregnancy test 16 days later.

### Statistical analyses

SPSS version 24 software (SPSS, Chicago, Illinois, USA) was used for analysis. Descriptive statistical analysis was described as median ± range. Multivariate Poisson regression was used to evaluate the effect of the aforementioned variables with the number of oocytes retrieved. Mann- Whitney *U* test was used to determine a relationship between the variables and their effect on OHSS. The Kruskal–Wallis test and Mann–Whitney *U* tests were used to determine the significance between age groups and probability of livebirth, based upon the age and number of oocytes retrieved. The Pearson’s Chi-squared test was used to determine statistical significance in those who returned to use their stored oocytes, and to determine the relationship between age at cryopreservation and successful livebirth. Statistical significance was set at *p* < 0.05. There has been no loss to follow up and where missing data is evident, this has been referred to in the results.

## Results

483 stimulation cycles were undertaken amongst 373 women. Table 1 summarises the demographic findings from the cohort of women included in the study.

**Table 1:** Demographic data and controlled ovarian stimulation variable

	Median (range)
Age (years)	38.3 (26–47)
BMI (Kg/m^2^)	22.5 (17.1–36.6)
FSH (IU/L)	7.2 (1.1–13.7)
AMH (pmol/l)	9.9 (0.17–56.6)
Baseline antral follicle count (*n*)	12 (2–49)
Number of days of stimulation (*n*)	9 (4–14)
Total dose of stimulation (IU)	3562.5 (700–13,875)
Antral follicles > 12 mm on day of trigger (*n*)	9 (1–50)
Oestradiol levels on day of trigger (per 1000 pmol/l)	7.01 (0.25–30.26)
Number of oocytes retrieved (*n*)	8 (0–37)
Number of metaphase II (*n*)	6 (0–28)
Number of metaphase I (*n*)	0 (0–8)
Number of germinal vesicles (*n*)	0 (0–19)

Univariable Poisson regression was used to assess the effect of each variable on oocyte yield (Table 2). This identified that age, baseline AFC, AMH, E2, and follicle count ≥ 12 mm on the day of trigger were all significant predictors of oocyte yield (*p* < 0.001).

**Table 2:** The incidence rate ratio (IRR), confidence interval (CI) and *p*-values to assess the effect of each variable on oocyte yield (univariate analysis)

	IRR	95% CI	*p*-value
Age (years)	0.92	0.91–0.93	< 0.001
BMI (kg/m^2^)	0.99	0.97–0.99	0.019
AFC (*n*)	1.39	1.03–1.04	< 0.001
AMH (pmol/l)	1.02	1.03–1.02	< 0.001
Oestradiol on day of trigger (per 1000 pmol/l)	1.08	1.07–1.09	< 0.001
Number of follicles > 12 mm on day of trigger (*n*)	1.04	1.04–1.04	< 0.001

*p value < 0.05 is statistically significant

Multivariable Poisson regression subsequently demonstrated that BMI (Incidence rate ratio; IRR; 1.02; 95% CI 1.00 − 1.03; 95% *p* = 0.04), E2 level per 1000 pmol/l increase (IRR 1.05; 95% CI 1.04 − 1.07; *p* < 0.001) and the number of follicles ≥ 12 mm at trigger (IRR 1.02; 95% CI 1.01–1.03; *p* < 0.001) were all significant predictors of oocyte yield (Table 3). However, there was no significant relationship between AFC (IRR = 1.00; 95% CI 0.99–1.02 *p* = 0.43) or AMH (IRR 1.00; 95% CI 0.99–1.01; *p* = 0.71) with oocyte yield. An increase in age of one-year resulted in a 4% reduction in oocyte yield after adjusting for all other variables (IRR 0.96; 95% CI 0.94–0.98; *p* < 0.001).

**Table 3:** The incidence rate ratio (IRR), confidence interval (CI) and *p*-values to assess the effect of each variable on oocyte yield (multivariate analysis)

	IRR	95% CI	*p*-value
Age (years)	0.96	0.94–0.98	< 0.001
BMI (kg/m^2^)	1.02	1.00–1.03	0.04
AFC (*n*)	1.00	0.99–1.02	0.43
AMH (pmol/l)	1.00	0.99–1.01	0.71
Oestradiol on day of trigger (per 1000 pmol/l)	1.05	1.04–1.07	< 0.001
Number of follicles > 12 mm on day of trigger (*n*)	1.02	1.01–1.03	< 0.001

*p value < 0.05 is statistically significant

There were nine (9/373; 2.4%) cases in total of OHSS; all of which were defined as mild in severity as per the Royal college of Obstetrician and Gynaecologist classification [16]. From these cases, women were administered with the following triggers: hCG (*n* = 4), Suprefact (*n* = 4) and dual (*n* = 1). There were no associated hospital admissions or significant adverse events. The Mann–Whitney *U* test identified that age, total AFC, number of follicles more than 12 mm on the day of trigger and FSH were all significant predictors of OHSS (*p* < 0.05). Whereas BMI, AMH and E2 levels however, were not significant predictors (Table 4).

**Table 4:** Mann–Whitney *U*, interquartile range (IQR) and *p*-values to assess the effect of each variable on oocyte yield on OHSS and non-OHSS groups

Ovarian reserve marker	OHSS median (IQR)	Non OHSS median (IQR)	*p* value
BMI (Kg/m^2^)	20.8 (5.75)	21.8 (4.0)	0.347
Age (years)	37.0 (3.5)	38.0 (4.0)	0.019*
Total AFC (*n*)	16.5 (22.0)	12.0 (8.0)	0.027*
AMH (pmol/l)	25.8 (25.5)	9.6 (10.9)	0.055
No follicles > 12 mm (*n*)	15.0 (11.0)	9.0 (7.0)	0.001*
FSH (IU/L)	6.1 (2.5)	7.3 (3.9)	0.006*
Oestradiol (per 1000 pmol/l)	123.0 (154.5)	147.8 (137.0)	0.361

*p value < 0.05 is statistically significant

Thirty-six women subsequently underwent 41 oocyte thaw cycles. The mean (SD) age at thaw was 42 (± 4.1). The mean number of years between cryopreservation and thaw was 3.7. Almost two-thirds (23/36; 63.9%) of women used their partner’s sperm, whereas just over a third (13/36; 36%) used donor sperm. Of the women who used donor sperm, the majority did so because they were single (9/13; 69%).

The percentage of oocytes which survived thaw was 81%, 76% and 67.5% in women aged ≤ 35, 36–39 and ≥ 40 years, respectively. The fertilization rate of the frozen thawed oocytes were 53%, 68% and 58% in the respective age groups. There were 37 embryo transfers (ET). Almost half (18/37; 48.6%) of the ET took place at the blastocyst stage. From the cycles with embryos cultured to the blastocyst stage, the average number of blastocysts formed were 55.6% (10/18), 45.5% (40/88) and 50% (9/18) in women aged ≤ 35, 36–39 and ≥ 40 years, respectively.

Following ET, 13 women had a confirmed clinical pregnancy, defined as the presence of a foetal heart visualised on ultrasound. The clinical pregnancy rate was 31.7% per thaw cycle. There were 12 livebirths achieved by 11 women, of which one woman delivered twins. Thus, the livebirth rate was 26.8% (11/41) per cycle and 29.7% (11/37) per ET. The remaining two women experienced a miscarriage, having both first cryopreserved their oocytes above 40 years old.

**Fig. 1:**
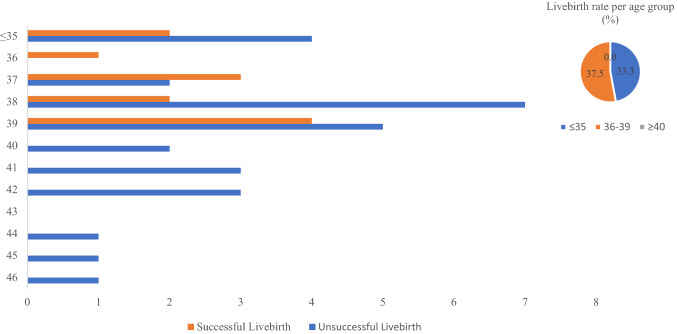
Number of thaw cycles per age group at first freeze (successful vs unsuccessful livebirth)

In women aged ≤ 35 years old, two livebirths from six oocyte thaw cycles were achieved, resulting in a livebirth rate per oocyte thaw cycle of 33%. Amongst women aged 36–39 years old, 24 cycles resulted in 9 livebirths. The livebirth rate was therefore 37.5% per oocyte thaw cycle. In those aged 40 years and over, all 11 cycles were unsuccessful, resulting in a livebirth rate of 0% (Fig. 1). The average age at cryopreservation of those who achieved a livebirth was 36.4, whereas those who did not was 39.0 (*p* = 0.07).

## Discussion

This study demonstrates herein that markers including E2, BMI and the number of follicles ≥ 12 mm at trigger, are all significant predictors of oocyte yield when controlling for confounding variables in women undergoing EOC. Whilst there are few studies assessing these relationships in the context of EOC, comparisons can be made from data from cycles performed for other indications, with consideration for differences in population characteristics.

AMH is considered a reliable predictor of oocyte yield [17]. However, it is well reported that levels are strongly correlated with age [18]. Although this study demonstrates AMH was a statistically significant predictor of yield from univariate analysis, there was no significant relationship when controlling for confounding variables with multivariate analysis. Such findings may be attributable to the fact that women undergoing EOC are young and fertile, unlike the vast majority of women undergoing in vitro-fertilisation (IVF). Considering AMH has been widely used as a marker of ovarian reserve in women undergoing assisted reproductive technology (ART), we can assume that the literature often refers to predominantly women with pre-existing diminished ovarian reserve [19]. Such cohorts include women who have undergone previous ovarian surgery or embarked on gonadotoxic treatment with chemotherapy or radiotherapy, and thus have reduced AMH levels irrespective of age [20]. Consequently, it is unsurprising that within infertile populations, evidence suggests AMH is an independent significant predictor of oocyte yield [21], whereas within our cohort of young healthy women, this was not demonstrated.  Our study highlights that age however, is a significant predictor of oocyte yield on multivariate analysis and therefore, perhaps a more reliable marker of ovarian reserve than AMH.

Studies suggest that oocyte yield is also associated with BMI [22]. In particular, oocyte quantity is lower in obese women when compared to those with a normal BMI [23]. Findings from this study however, contrarily demonstrate that oocyte yield is increased by 2% per increase of BMI measured (kg/m^2^). The majority of women in our cohort had a normal BMI 20.0–24.9 (*n* = 111), less were categorised as being underweight (BMI < 20.0, *n* = 34) and very few were obese (BMI > 30.0, *n* = 9). In contrast to previous literature, the mean number of oocytes collected in the obese group was higher than the underweight, although not to a significant extent (9.6 vs 8.5). This further implies therefore, that in the context of EOC, patient demographics are dissimilar to the standard IVF population. Obese infertile women may have associated subfertility related to their body habitus, such as those with polycystic ovarian syndrome; however, overweight fertile women may not have similar discernible reduction in oocyte yield. This is exemplified by previous studies where obese women contributed to 8.9% of the total cohort, compared to only 2.9% in our own study [24]. As such, much of the evidence on prediction of oocyte yield deduced from IVF cycles in infertile women may not be extrapolatable to EOC populations.

At the time of writing, 9.7% of women who underwent EOC had returned to use their oocytes, a mean 3.7 years following cryopreservation. A study reporting outcomes from 1382 women reported a utilisation rate of 8.7% (*n* = 120) after 2.2 years of cryopreservation [25]. Furthermore, amongst a sample of 254 women in Sweden, the utilisation rate was 15% (*n* = 38) on average 4 years following cryopreservation [26], whilst another survey reported a rate of 6% from 96 women 2.8 years after EOC [27]. Low utilisation rates may be attributed to the fact that women less than 35 years old may conceive spontaneously, by virtue of their young age at cryopreservation.

The clinical pregnancy rate in our study was 31.7% at a median age of 43 years old. This is comparable to the average pregnancy rates reported by the Human Fertilisation and Embryology Authority (HFEA) in women aged between 35 and 37 years old [28]. Such findings epitomise the reproductive potential of EOC and provides further clinical correlation of the concept. Furthermore, just under a third (30.5%) of women who returned to use their stored oocytes attained a livebirth. This is favourable compared to a recent study analysing 10 years of data from two UK clinics who had a success rate pertaining to livebirths or ongoing pregnancies of 17.5% [29]. Just over a third (37%) of women aged < 40 years old in our study achieved a livebirth or ongoing pregnancy. This is higher than the reported successful outcomes of 20–23% reported in the aforementioned study [29]. In a Swedish study publishing outcomes from 38 women following EOC, the cumulative live birth rate was 63%, 26% and 0% in women of ages 36–37, 38–39 and ≥ 40 years at vitrification, respectively [26], compared to live birth rates of 33% (*n* = 6), 37.5% (*n* = 24), and 0% (*n* = 11) in women aged ≤ 35, 36–39 and ≥ 40 years old, respectively in our own study.

All women who underwent EOC ≥ 40 years old and returned to thaw their oocytes had an unsuccessful reproductive outcome. Almost three quarters (72.7%) had also exhausted their supply of stored oocytes following attempt of oocyte thaw. This proportion is more than double that reported following assessment of outcomes from the aforementioned Swedish study, whereby no successful livebirths were achieved in 11 women who underwent EOC over the age of 40, and 36% (*n* = 4) had exhausted their oocyte supply [26]. The previously discussed UK study across two centres fared slightly better, with 7% of those aged 40–42 having a successful outcome [29]. Whilst the overall numbers remain small, it is clear undergoing EOC aged ≥ 40 years is unlikely to result in successful reproductive outcomes. Not only are the success rates low, but the risk of miscarriage remains high, as exemplified by the fact the two women who suffered a miscarriage in our study were both aged > 40 years old. This supports evidence which suggests the risk of miscarriage in women aged 40–44 is 51% [30].

From an economic perspective, EOC may not be a cost-effective procedure for women who undergo cryopreservation ‘too early,’ for example in their twenties, due to the likelihood of returning to stored oocytes being low and the chances of successful conception being high. Younger women may expose themselves to unnecessary physical risk and substantial financial burden given the expense of longer oocyte storage required. [31] Based on a cost-effective analysis, it has been deduced that the optimal age to undergo oocyte freezing is 35 years old, assuming a probability of returning to cryopreserved oocytes of > 61% and the willingness to spend approximately €19,560 per livebirth [32]. Evidently, each case should be individually considered, with appropriate contemplation of the physiological, obstetric, legal and economic considerations [33].

The findings of this study add substantially to published literature to identify factors that impact success following EOC. Data with long term follow up is imperative, so that women can be appropriately counselled regarding their probability of oocyte yield following ovarian stimulation, and subsequent success of livebirth. Whilst speculative, if women in this cohort were aware they had a very low chance of success, they may have undergone further cycles to optimise their future chances, or indeed if are 40 years and above, not embark upon treatment in the first place. Other studies have shown that women are willing to undergo two or more cycles in order to retrieve sufficient numbers of oocytes for storage [34]. Therefore, it is essential women have the most accurate individualised estimate of their likelihood of future success, in order to make informed choices regarding number of cycles and facilitate the management of future expectations. However, it is paramount that no matter how high the predicted percentage chance of achieving a livebirth is, women are aware the likelihood of a livebirth cannot be guaranteed. This is important given evidence has suggested women who overestimate expectancy of livebirth, or have few oocytes to preserve, often regret oocyte cryopreservation, resulting in subsequent emotional and psychological sequalae [35].

Strengths of this study include that it is one of the largest cohort analyses with long duration of follow up. Albeit, no longer a novel method of fertility preservation, this study is the first to describe clinical outcomes specific to EOC, such as the incidence of OHSS, success of autologous oocyte thaw cycles and live birth rates. Limitations include the retrospective nature of data analysis from a single centre and the small sample size regarding reproductive outcomes. This is attributed to the low utilisation rate of cryopreserved oocytes, which is consistent worldwide. Moreover, the decision to give either GnRH antagonist or dual trigger were made on an individual basis following clinical judgment, including consideration of previous response to stimulation along with baseline AMH and AFC and E2 levels on the day of trigger. The lack of strict criteria thus, further introduces the potential for bias in this analysis, as well as the lack of control for confounding variables, which may affect oocyte yield, such as pre-existing conditions affecting fertility. All of the above could be minimised by the use of a multi-centre randomised prospective study.

## Conclusion

This study highlights that age, BMI, E2, and number of follicles ≥ 12 mm at trigger can predict oocyte yield in EOC cycles. As such, individualising EOC cases with consideration of these markers should be undertaken to optimise oocyte yield. AMH and AFC are not, however, significantly associated with oocyte yield. This is the first study to establish an incidence of OHSS amongst EOC cycles and establish a significant relationship between age, total AFC, number of follicles ≥ 12 mm and FSH as predictors of OHSS. Moreover, the reproductive outcomes presented highlight that undertaking EOC over the age of 40 years old is suboptimal and associated with low success rates of livebirth. Whilst there was no significant difference in livebirth rates in women ≤ 35 years and aged 36–39, it is advisable that EOC should be undertaken at, or prior to the age 36 to optimise reproductive outcomes. This data has significant implications for clinical practice, and can be used to individualise care, enhance counselling and manage expectations in women undergoing EOC.
